# The impact of scaffolded and non-scaffolded suicidal virtual human interaction training on clinician emotional self-awareness, empathic communication, and clinical efficacy

**DOI:** 10.1186/s12909-024-05371-9

**Published:** 2024-04-15

**Authors:** Heng Yao, Alexandre Gomes de Siqueira, Megan L. Rogers, Sarah Bloch-Elkouby, Olivia Lawrence, Giuseppe Sarli, Adriana Foster, Serge A. Mitelman, Igor Galynker, Benjamin Lok

**Affiliations:** 1 Department of Computer and Information Science and Engineering, University of Florida, Gainesville, 32611 FL USA; 2 Department of Psychology, Texas State University, San Marcos, 78666 TX USA; 3 Department of Psychiatry and Behavioral Sciences, Icahn School of Medicine at Mount Sinai, New York, 10029 NY USA; 4 Healthcare Corporation of America (HCA) Florida, Woodmont Hospital, Tamarac, 33321 FL USA; 5 Ferkauf Graduate School of Psychology, Yeshiva University, New York, 10033 NY USA; 6 Department of Mental Health and Addiction, ASST Brianza, Vimercate, Italy

**Keywords:** Suicidal virtual patient, Scaffolded instructions, Verbal empathic communication training, Negative emotional responses, Clinical efficacy

## Abstract

**Background:**

Clinicians working with patients at risk of suicide often experience high stress, which can result in negative emotional responses (NERs). Such negative emotional responses may lead to less empathic communication (EC) and unintentional rejection of the patient, potentially damaging the therapeutic alliance and adversely impacting suicidal outcomes. Therefore, clinicians need training to effectively manage negative emotions toward suicidal patients to improve suicidal outcomes.

**Methods:**

This study investigated the impact of virtual human interaction (VHI) training on clinicians’ self-awareness of their negative emotional responses, assessed by the Therapist Response Questionnaire Suicide Form, clinicians’ verbal empathic communication assessed by the Empathic Communication and Coding System, and clinical efficacy (CE). Clinical efficacy was assessed by the likelihood of subsequent appointments, perceived helpfulness, and overall interaction satisfaction as rated by individuals with lived experience of suicide attempts. Two conditions of virtual human interactions were used: one with instructions on verbal empathic communication and reminders to report negative emotional responses during the interaction (scaffolded); and the other with no such instructions or reminders (non-scaffolded). Both conditions provided pre-interaction instructions and post-interaction feedback aimed at improving clinicians’ empathic communication and management of negative emotions. Sixty-two clinicians participated in three virtual human interaction sessions under one of the two conditions. Linear mixed models were utilized to evaluate the impact on clinicians’ negative emotional responses, verbal empathic communication, and clinical efficacy; and to determine changes in these outcomes over time, as moderated by the training conditions.

**Results:**

Clinician participants’ negative emotional responses decreased after two training sessions with virtual human interactions in both conditions. Participants in the scaffolded condition exhibited enhanced empathic communication after one training session, while two sessions were required for participants in the non-scaffolded condition. Surprisingly, after two training sessions, clinical efficacy was improved in the non-scaffolded group, while no similar improvements were observed in the scaffolded group.

**Conclusion:**

Lower clinical efficacy after virtual human interaction training in clinicians with higher verbal empathic communication suggests that nonverbal expressions of empathy are critical when interacting with suicidal patients. Future work should explore virtual human interaction training in both nonverbal and verbal empathic communication.

## Background

In the United States, suicide rates increased by approximately 36% between 2000 and 2021 [1]. In 2021, suicide accounted for 48,183 deaths, ranking among the top nine leading causes of death for individuals aged 10-64 and as the second leading cause of death among those aged 10-14 and 20-34 in the US [1]. Clinicians are key players in preventing suicide, yet they often struggle to manage their negative emotional responses (NERs) and engage in less empathic communication (EC) when faced with a patient at risk of suicide [2, 3]. Clinicians’ NERs, such as distress, detachment, and hopelessness, toward suicidal patients after they express suicidal ideation (SI) affect the patients’ short- and long-term outcomes. Distress is a general unpleasant feeling, including worry, sadness, and pain; detachment is an inner barrier to connecting to the patient or an urge to end the interview; hopelessness is a lack of hope for the patient because nothing would prevent his or her suicide [4–6]. NERs and deficits in EC significantly impair the therapeutic alliance, increasing the risk of suicidal outcomes [4, 7]. Researchers recognize empathy as a fundamental component in delivering superior patient care [8]. Empathic skills are essential for clinicians to form therapeutic relationships, which are fostered by trust and open communication about emotional states [9]. Additionally, NERs detrimentally influence the decision-making process in clinical settings, potentially resulting in critical misjudgments like inappropriate emergency discharges or unnecessary compulsory hospitalizations, thereby undermining effective psychiatric care [10, 11]. Therefore, it is essential for clinicians to be trained in the management of NERs and engagement in EC to ensure effective suicide prevention.

Recent advances in virtual human technology have enabled the development of interactive tools to train clinicians in effective communication skills when engaging with suicidal patients [12–15]. Empathic opportunities were deliberately incorporated into virtual patient interactions to evoke empathic reactions from users. Empathic opportunities were crafted based on Bylund and Makoul’s definition, which describes them as “patient statements that present an explicit expression of emotion, progress, or challenge by the patient [16, 17]”. Bylund et al. proposed the Empathic Communication and Coding System (ECCS) to assess the level of empathy in responses to empathic opportunities [16, 17]. Virtual patient interactions were used to enhance medical students’ empathic communication skills [12, 18, 19]. Kleinsmith et al. discovered that virtual patients provide a less stressful interaction environment, making them effective tools for training medical students in empathic communication skills [12]. Researchers found that feedback on empathy during interactions with virtual patients enhanced medical students’ empathic responses in subsequent encounters with standardized patients [18, 19]. These studies highlight the promising role of virtual humans in training verbal empathic communication skills. They achieve this by providing a coding of users’ empathic responses using the ECCS-scale at the end of the interaction [18, 19].

Providing trainees with summative feedback at the end of a virtual human encounter has a limited impact on training, particularly in assisting trainees in formulating responses that acknowledge the central issue and validate the feelings expressed by patients [20]. Issenberg et al. highlighted key features of effective medical simulations, which include feedback during the learning experience, repetitive skill practice, and progressively more challenging scenarios [21]. Similarly, Bosse et al. demonstrated that both high- and low-frequency intermittent feedback significantly enhances students’ early skill acquisition, with high-frequency feedback leading to smoother [22]. Integrating instructions at various points offers immediate opportunities for corrective action, aligning with Vygotsky’s principles of proximal development [23]. The zone of proximal development (ZPD) defines the gap between what a trainee can do alone and what they can achieve with guidance. Effective instruction occurs within this zone, presenting tasks just beyond the trainee’s current capabilities to promote cognitive growth [23]. Educators are advised to provide scaffolding, supportive activities or peer assistance, to help trainees navigate through the ZPD [24]. Wells described scaffolding as operationalizing Vygotsky’s concept of the ZPD [23, 25]. Modern educational approaches see scaffolding as strategies and guides used by educators and technology to help learners achieve understanding beyond their current capacity [26]. This process involves gradually reducing support as trainees demonstrate mastery, effectively transferring the learning responsibility from the trainer to the trainee [27, 28]. This method leverages deep retrieval processes for generating responses from memory [29, 30]. Finn et al. found that scaffolded feedback leads to more resilient corrections over time than standard feedback [31].

### Aims

Virtual human interactions provide a low-pressure environment where users can reflect on their responses, fostering the development of empathy, emotional self-awareness, and other communication skills crucial for identifying and managing suicide risks [12, 18, 19]. Building on these promising results, this research project has developed virtual human interactions integrated with scaffolded instructions to complement traditional training in descriptive suicide risk assessment (e.g., identifying suicidal ideation, planning, intent, and other risk factors). Our aim is to enhance clinicians’ awareness of NERs and their skills in verbal EC, thereby improving clinical efficacy and strengthening the therapeutic alliance. Ultimately, our goal is to foster authentic and supportive encounters with suicidal patients, contributing to life-saving interventions.

This study aimed to investigate the impact of using VHIs integrated with scaffolded instructions on enhancing EC and reminders to report NERs, to improve clinician outcomes. The study assessed outcomes from pre- (T1) to post-training (T2 and T3) and compared results between VHI training sessions with and without scaffolded instructions and reminders integrated into the interaction. The three hypotheses were that while both VHI training methods would be impactful, scaffolded training would outperform non-scaffolded training in three key areas: reducing clinicians’ negative emotional responses (NERs) toward virtual patients, enhancing empathic communication (EC) with virtual patients, and improving overall clinical efficacy in interactions with virtual patients, as evaluated by raters with lived experience of suicide attempts.

## Methods

### Context and participants

This study was part of a multi-site project conducted at FIU/Citrus Health Network in Florida, Mount Sinai Beth Israel Hospital in Manhattan, and Elmhurst Hospital in New York City from 2020 to 2022. The goal was to assist clinicians in managing their NERs towards suicidal patients and improve outcomes using virtual human interaction technologies. We conducted a quasi-experimental study, providing a 12-week training session that included two groups of participants, each interacting with three VHIs.

In the first year (2020-2021), 27 participants were recruited for the experimental condition, which included scaffolded instructions to enhance empathic communication (EC) and reminders to report NERs, integrated into the first two VHIs. Some of the data collected in this condition were previously reported in Yao et al. [32]. In the second year (2021-2022), 35 participants were recruited for the control group, where no scaffolded instructions or reminders were integrated into the VHIs.

A total of 62 participants from the behavioral health field were engaged in this study, including trainees and staff from FIU/Citrus Health Network in Hialeah, FL, Mount Sinai Beth Israel Hospital in Manhattan, and Elmhurst Hospital in New York City. The diverse group of participants included psychiatry residents (second to fourth years), child and adolescent psychiatry fellows, advanced psychology interns, Ph.D. candidates in clinical psychology, psychology postdoctoral fellows, master’s students in their final year of mental health counseling and social work, and licensed clinicians (including psychiatrists, psychologists, social workers, and mental health counselors) across these sites. Results of a sensitivity power analysis indicated that, with a sample size of 62, alpha of .05, and power of .80, we were adequately powered to detect small effect sizes (Cohen’s f-squared = .0256) in our interaction (time by condition) analyses.

To avoid potential coercion, study investigators, who were faculty in the respective programs and directly supervised potential participants, did not partake in recruitment. Participants received $50 upon completion of the study. The team of investigators included six individuals with lived experience of suicide attempts, who contributed by rating clinicians’ performances. This inclusion aimed to empower the lived experience community in enhancing treatment approaches for patients at risk of suicide.

### Virtual human interaction training

In this study, we adapted three existing education interventions. In the training intervention, three virtual patients, Cynthia Young, Bernie Cohen, and Denise Jones, were used. We describe different components that are related to the design and development of the virtual human training system, including: 1) virtual patient scenarios, 2) virtual human interactions, and 3) scaffolded instructions.

#### Virtual patient scenarios

*Cynthia Young* virtual patient scenario was created and validated by Shah et al. [33]. The scenario was used in various research and educational applications [19, 32, 34, 35]. Cynthia, a 21-year-old college student referred by her campus counselor, presents to the doctor with depression. She developed passive suicidal thoughts after a personal loss (her cousin was killed in a car accident eight months earlier). She failed two courses in the past semester because she could not wake up on time or concentrate well enough to study. She quit her job in a bookstore because she no longer cares about books, stopped volunteering for a local charity, and now spends a lot of time alone in the apartment, which is not typical for her [18, 33].

*Bernie Cohen* virtual patient scenario was created and validated by our team from a patient case published by Galynker et al. [35, 36]. The scenario was used and validated in Yao et al. [32, 35]. Bernie is a 53-year-old gay man with a history of generalized anxiety disorder who developed suicidal ideation and suicide plans after unexpectedly losing his partner. Bernie came to see a psychiatrist at the suggestion of a friend due to depression with suicidal ideation after he discovered that his just deceased partner of 20 years had a wife and young children [36].

*Denise Jones* virtual patient scenario was created by Foster et al. and is available on MedEdPORTAL with instructions to use by educators [37]. Denise is a 43-year-old woman with three children at her first visit to a psychiatrist. Her only past psychiatric history involved treatment with medication by a primary care practitioner four years ago. Denise has just quit her job in the past week, and she cannot sleep. She has paranoia and grandiosity and is impulsive [37, 38].

#### Virtual human interactions

**Fig. 1:**
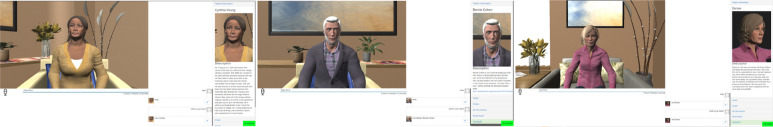
Virtual patients used. Cynthia Young (left), Bernie Cohen (middle), and Denise (right)

VHIs include a review of descriptive suicide risk assessment elements based on APA Guidelines [39], and the proposed DSM-5 criteria for Suicidal Crisis Syndrome before the actual interaction [5, 36]. Each VHI was followed by the TRQ-SF. Virtual human interactions are hosted using Virtual People Factory (VPF), previously utilized to create interventions that improve clinician-patient communication [40, 41]. VPF can process users’ input, including typed phrases and spoken phrases, and apply a variety of algorithms to determine which set of virtual patient responses (i.e., verbal response) is a paraphrase of any of the virtual human’s structured set of questions and answers [41]. All similarity measures are provided to a machine-learning model that classifies the likelihood that an input question is a paraphrase [41]. As VPF is modular, the algorithms that VPF uses can be interchanged and collectively applied. VPF uses both information retrieval algorithms (in which predefined input is most similar to the user’s input) and intent recognizers such as machine learning (Google’s DialogFlow [42]) and natural language processing systems (Microsoft LUIS [43]). The VHI interaction took place in the Unity3D game engine, presenting highly realistic animated virtual humans to the user. Figure 1 shows the virtual suicidal patients used in the study.

#### Scaffolded instructions

We integrated real-time instructions into the interaction to remind participants how to express empathy verbally and to remind them to reflect on their negative emotions. During the interactions with Cynthia and Bernie, the first five empathic opportunities were marked by a dialog box. Participants were asked to interact with Cynthia first and Bernie second. As participants progressed in the study, they received reduced levels of guidance. As such, there is less information in the dialog in the interaction with Bernie than with Cynthia, as participants were expected to respond to empathic opportunities by themselves.

**Fig. 2:**
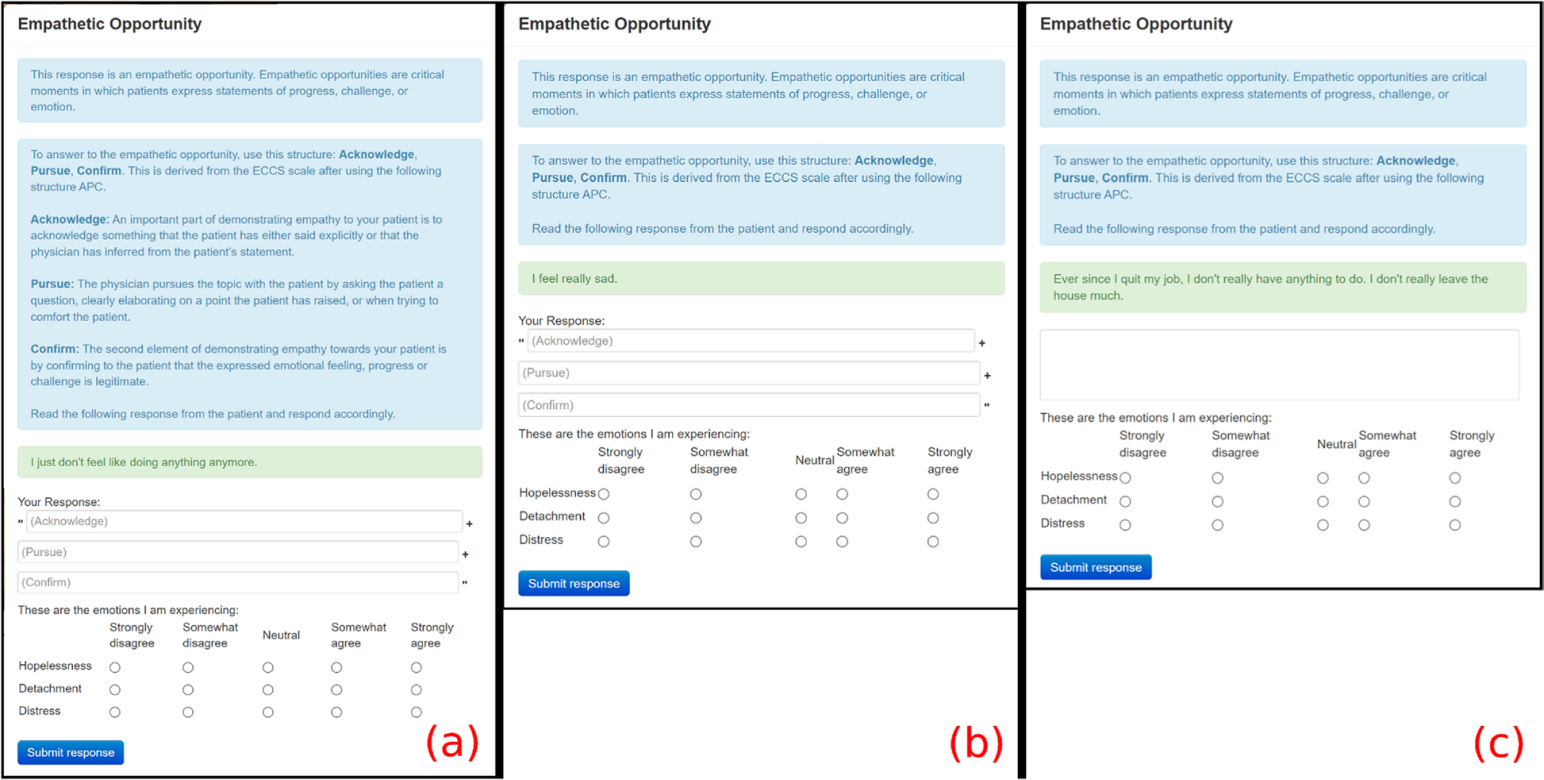
Scaffolded instructions and reminders to report NERs in the interaction with Cynthia

**Fig. 3:**
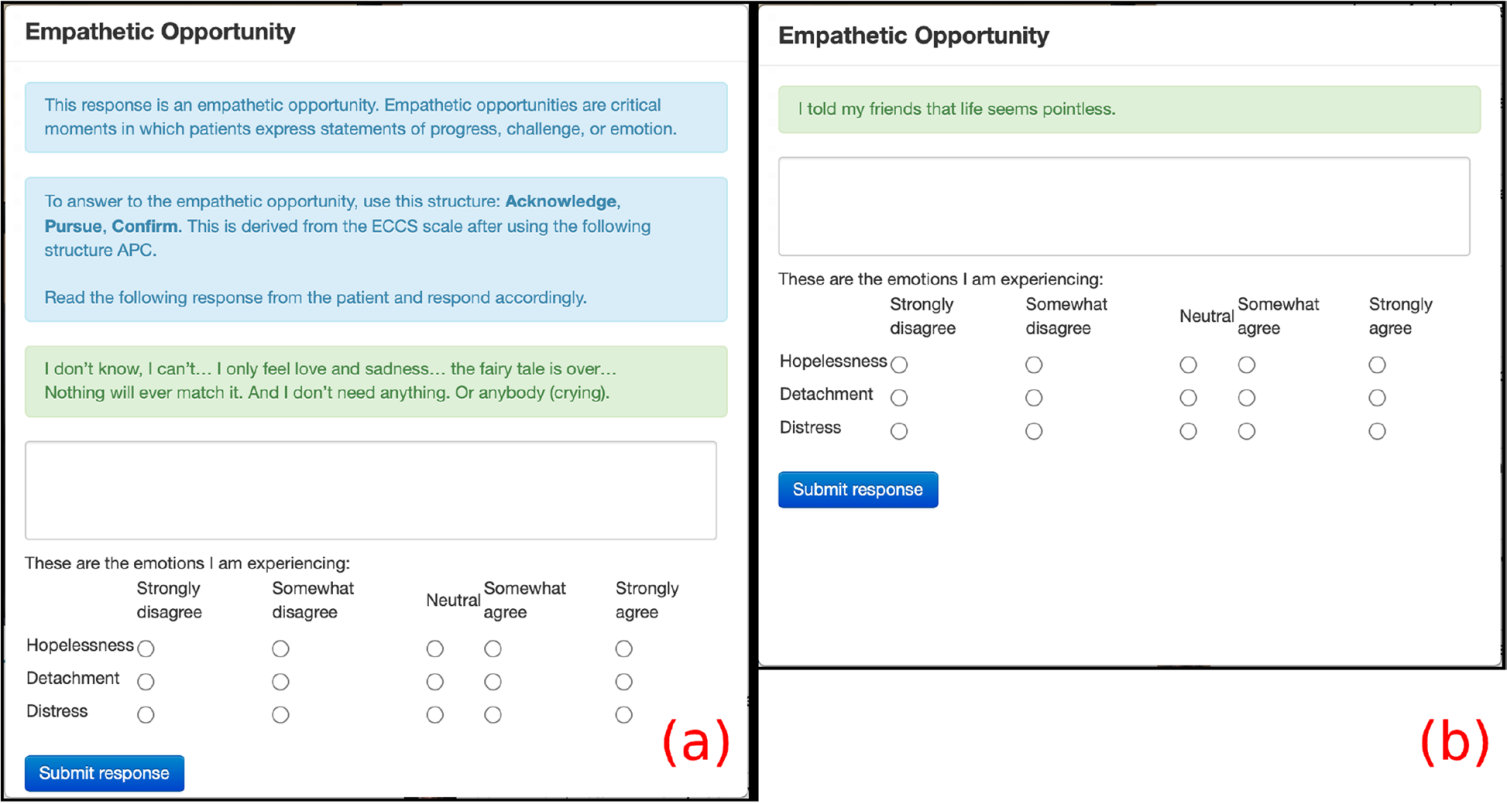
Scaffolded instructions and reminders to report NERs in the interaction with Bernie

We emphasized the structure of the APC scaffolding (Acknowledge, Pursue, Confirm) based on the ECCS scale for participants to formulate empathic responses [16, 17]. In Cynthia’s first empathic opportunity, participants were presented with a detailed explanation of APC, as shown in Fig. 2a. In Cynthia’s two subsequent opportunities and Bernie’s first two opportunities, a shorter explanation of APC appears to the participants, as shown in Figs. 2b and 3a. In the following two (i.e., fourth and fifth) empathic opportunities of Cynthia and the next three (i.e., third, fourth, and fifth) empathic opportunities of Bernie, a dialog without APC appears to the participants, as shown in Figs. 2c and 3b. There were no pop-up dialogs in the interaction with Denise to evaluate the endurance of the training.

#### VPF and human empathy rating in VHIs

VPF fed the study participants’ responses into a machine learning classifier, which is trained on numerous users’ responses to virtual humans [35]. Through this feedback system, the VPF is tuned to how participants will react and empathize with virtual patients and identify an empathic response level along the Empathic Communication Coding System-ECCS [12, 16–18]. As a result, the clinician participants received feedback on their empathic responses to the opportunities presented by the virtual humans after the interaction.

In addition to the automated ECCS coding in this study, the responses of the clinicians to the predetermined empathic opportunities found in the transcripts were expertly rated with ECCS by the study investigators to validate the feedback of the VHI. The investigators underwent training in ECCS coding to achieve a final interrater reliability of =0.8 measured by intra-class correlation.

### Study procedure

**Fig. 4:**
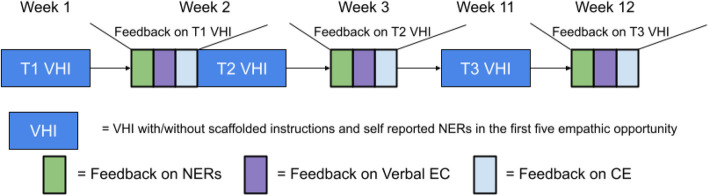
Study procedure to complete three virtual human interactions

All participants in the study engaged in three VHIs over a 12-week period. The first two VHIs were scheduled at 1-week intervals (one VHI per week at T1 and T2), with feedback for VHI 1 given immediately before VHI 2. Feedback for VHI 2 was provided one week after its completion. Two months later, participants undertook VHI 3 and received feedback one week afterwards. Figure 4 illustrates the detailed procedure for completing the three VHIs and receiving feedback for each. Before each interaction with a virtual patient, participants were shown an introduction page. This page included videos that introduced empathic opportunities, provided background information on the virtual patient, outlined the goals of the interaction, and offered instructions on how to use the system. During the interactions, participants were able to ask the virtual patients questions, with the empathic opportunities specifically designed to elicit an emotional response.

In the experimental condition, the first five empathic opportunities during interactions with Cynthia and Bernie were highlighted by a dialog box (refer to Figs. 2 and 3). These dialog boxes provided scaffolded instructions for responding to empathic opportunities and reminded participants to reflect on their negative emotions. As participants progressed, they received gradually reduced guidance, with the dialog boxes during interactions with Bernie containing less information than those with Cynthia. After the first five empathic opportunities, no further dialog boxes were presented, expecting participants to independently manage their responses to empathic opportunities and their NERs. In contrast, the control group received no scaffolded instructions or reminders to reflect on NERs for any of the VHIs.

Following each VHI, clinician participants (CPs) in both the experimental and control conditions received detailed feedback on three key aspects: negative emotions, verbal empathic communication, and clinical efficacy. This feedback included an analysis of the participants’ self-reported negative emotions, providing guidance on understanding and managing these emotions. It also evaluated CPs’ empathic responses to the predetermine empathic opportunities, automatically assessed by the VPF software. Regarding clinical efficacy, feedback included ratings on a 5-point Likert scale, assessing the impact of the interaction on patient willingness to return for follow-up appointments and the perceived helpfulness in reducing suicidal intent. It also provided interpretative comments from co-investigators, especially highlighting areas for improvement based on the efficacy, empathy, or alliance ratings. Such feedback suggested focusing on improving empathy and alliance to enhance clinical efficacy. Additionally, CPs (Clinician Participants) gained insights into their performance through the Medical Interview Satisfaction questionnaire, which was completed by the raters. This scale assesses Communication Comfort, Distress Relief, Compliance Intent, and Rapport, with the goal of enhancing their skills in clinical interactions [44].

### Data analytic strategy

Descriptive statistics were first computed for the full sample and stratified by (1) time-point (i.e., T1, T2, and T3) and (2) study condition (i.e., scaffolded instructions and non-scaffolded instructions) on each relevant outcome variable: clinicians’ negative emotional responses, clinicians’ empathic responses, and clinicians’ efficacy (i.e., patient’s likelihood of returning for follow-up appointments, perceived helpfulness in reducing suicidal intent, and interview satisfaction, as rated by individuals with lived experience). Linear mixed models were then computed to examine changes in these outcomes over time, as moderated by study condition (i.e., scaffolded versus non-scaffolded feedback) [45]. Specifically, the main and interaction effects of time-point and study condition were entered as fixed predictors, whereas clinicians’ negative emotional responses, empathic responses, and efficacy were entered as outcome variables in separate models. Random intercepts for clinicians were entered in all models to account for the non-independence of observations. Clinician characteristics, including age, gender identity, and degree type, were included as covariates. Analyses were conducted in R using the lme4 [46], lmerTest [47], and emmeans packages [48]. Missing data were minimal and addressed with listwise deletion.

The three hypotheses were that while both VHI training methods would be effective, the scaffolded training would be superior to non-scaffolded training in reducing clinicians’ NERs toward virtual patients, improving EC when communicating with virtual patients, and improving overall clinical efficacy when interacting with virtual patients as rated by raters with lived experience of suicide attempts.

### Measures

#### Negative emotional responses (NER)

Our first hypothesis, that scaffolded training would be superior to non-scaffolded training in reducing clinicians’ NERs toward virtual patients, was tested using the Therapist Response Questionnaire-Suicide Form (TRQ-SF). This tool, a 10-item Likert-type scale, is designed to measure NERs, capturing clinicians’ emotional responses to acutely suicidal patients with items such as ‘I had to force myself to connect’ and ‘I felt my hands were tied or put in an impossible bind’. TRQ-SF individual item scores range from 0 (Not at all) to 4 (Extremely) [4, 5]. The questionnaire comprises three factors: affiliation, distress, and hopefulness, which load onto a single common higher-order factor of general negative valence emotional response; positive valence items are reverse scored on this factor, with high internal reliability (alpha =.88) [4, 6]. The potential range for TRQ total scores is 0-40. TRQ-SF scores were associated in both concurrent and predictive ways with patient suicidal outcomes [4, 5], depression severity, and clinicians’ judgment of patient suicide risk, but not with global symptom severity, thus specifically indicating suicide-related responses [4].

#### Empathic communication (EC)

Our second hypothesis posited that scaffolded training would be superior to non-scaffolded training in enhancing empathic communication (EC) with virtual patients. To test this, we employed the Empathic Communication Coding System (ECCS), a tool developed to identify empathic opportunities and assess clinicians’ verbal responses to these moments [16, 17]. The ECCS codes healthcare providers’ verbal responses to empathic opportunities ranging from level 6 (clinician shares the patient’s emotion) to level 0 (clinician ignores the empathic opportunity or denies the patient’s perspective) [16, 17]. Our team has used ECCS extensively to code empathic responses in VHI transcripts [18, 32, 35, 49, 50].

#### Clinical efficacy (CE)

Our third hypothesis posited that scaffolded training would outperform non-scaffolded training in boosting overall clinical efficacy. This efficacy was evaluated in terms of the likelihood of patients returning for follow-up appointments, perceived helpfulness in reducing suicidal intent (assessed on a 5-point Likert scale), and overall interview satisfaction (measured by the Medical Interview Satisfaction questionnaire). Co-investigators with lived experience of suicide attempts conducted these evaluations. After examining both the interaction transcript and the recorded video, which focused on the participants’ facial expressions, raters provided feedback in a free text section, outlining the strengths and weaknesses of the trainee’s performance during the VHI.

## Results

### Trainees’ negative emotional responses

Hypothesis 1 assessed if scaffolded training was more effective than non-scaffolded training in reducing clinicians’ negative emotional responses (NERs) from the start of training (T1) to follow-up points (T2 and T3). Results indicated a significant reduction in NERs from T1 to T3 (B = -2.50, SE = 1.02, *p* = 0.016), but no significant change from T1 to T2 or differences between training conditions (B = -0.30, SE = 1.31, *p* = 0.817). There were also no significant interaction effects between time and condition (B = -0.27 and 0.42, SE = 1.42 and 1.43, *p* = 0.847 and 0.771), suggesting no variance in NER reduction between scaffolded and non-scaffolded groups over time. This finding did not support Hypothesis 1. Covariates did not significantly predict NERs, with p-values ranging from 0.061 to 0.995. Detailed regression analysis results are presented in Table 1.

**Table 1:** Multilevel regressions testing the relationship between Time and Condition to NERs to a virtual patient

Variable	B	SE	*P*	95% CI
**Fixed Effects**				
(Intercept)	16.26	3.28	<0.001	[9.71, 22.81]
Clinician Age	-0.001	0.08	0.995	[-0.17, 0.17]
Cisgender Woman	-1.01	1.03	0.329	[-3.04, 1.06]
Clinician Degree: Staff MD/DO	-1.30	1.68	0.445	[-4.66, 2.05]
Clinician Degree: Staff Psychologist	-1.98	2.12	0.355	[-6.24, 2.25]
Clinician Degree: MSW/MHC/MFT	-4.59	2.40	0.061	[-9.37, 0.20]
Clinician Degree: Trainee Psychiatrist	0.59	1.79	0.742	[-2.97, 4.16]
Clinician Degree: Trainee Psychologist	0.11	1.95	0.956	[-3.81, 4.00]
Clinician Degree: Masters Clinician	-4.34	2.59	0.098	[-9.48, 0.80]
Clinician Degree: Other	0.46	2.27	0.839	[-4.08, 4.99]
Study Condition - Non-Scaffolded	-0.30	1.31	0.817	[-2.90, 2.29]
Time - T2	-1.16	1.01	0.251	[-3.17, 0.83]
**Time - T3**	**-2.50**	**1.02**	**0.016**	**[-4.53, -0.48]**
Non-Scaffolded x T2	0.42	1.43	0.771	[-2.42, 3.26]
Non-Scaffolded x T3	-0.27	1.42	0.847	[-3.08, 2.54]
**Random Effects**				
$\tau _{00,subject}$	6.5			
$\sigma ^{2}$	11.24			
$N_{subject}$	52			
Observations	137			
Deviance	767.5			

Results that are statistically significant are highlighted in bold

**Table 2:** Multilevel regressions testing the relationship between Time and Condition to Verbal EC to virtual patients

Variable	B	SE	*P*	95% CI
**Fixed Effects**				
(Intercept)	2.46	0.41	<0.001	[1.65, 3.28]
Clinician Age	-0.002	0.01	0.816	[-0.02, 0.02]
**Cisgender Woman**	**0.36**	**0.12**	**0.004**	**[0.12, 0.60]**
Clinician Degree: Staff MD/DO	-0.07	0.20	0.720	[-0.46, 0.32]
Clinician Degree: Staff Psychologist	-0.05	0.25	0.842	[-0.54, 0.44]
Clinician Degree: MSW/MHC/MFT	0.07	0.29	0.811	[-0.50, 0.65]
Clinician Degree: Trainee Psychiatrist	0.06	0.21	0.786	[-0.36, 0.47]
Clinician Degree: Trainee Psychologist	-0.05	0.22	0.840	[-0.49, 0.41]
**Clinician Degree: Masters Clinician**	**-0.66**	**0.31**	**0.038**	**[-1.28, -0.04]**
Clinician Degree: Other	0.05	0.27	0.838	[-0.49, 0.58]
ECCS: Number of Opportunities	-0.03	0.01	<0.001	[-0.05, -0.02]
ECCS: Duration of Interaction	0.02	0.01	0.042	[0.001, 0.04]
Study Condition - Non-Scaffolded	-0.11	0.19	0.544	[-0.49, 0.26]
**Time - T2**	**0.53**	**0.16**	**0.002**	**[0.20, 0.85]**
**Time - T3**	**0.60**	**0.17**	**<0.001**	**[0.25, 0.94]**
**Non-Scaffolded x T2**	**-0.51**	**0.23**	**0.026**	**[-0.96, -0.06]**
Non-Scaffolded x T3	-0.33	0.23	0.160	[-0.79, 0.13]
**Random Effects**				
$\tau _{00,subject}$	0.03			
$\sigma ^{2}$	0.31			
$N_{subject}$	52			
Observations	143			
Deviance	251.1			

Results that are statistically significant are highlighted in bold

### Trainees’ verbal empathic communication (EC)

Hypothesis 2 explored the effects of scaffolded versus non-scaffolded VHI training on clinicians’ verbal empathic communication (EC) from pre-training (T1) to post-training assessments (T2 and T3). Significant increases in EC were observed across the entire sample from T1 to T2 (B = 0.53, SE = 0.16, *p* = 0.002) and T1 to T3 (B = 0.60, SE = 0.17, *p* < 0.001). A significant interaction between Time and Condition was observed in predicting changes in empathic responses from T1 to T2 (B = -0.51, SE = 0.23, *p* = 0.026), though this pattern did not persist from T1 to T3 (B = -0.33, SE = 0.23, *p* = 0.160). Specifically, improvements in empathic communication (EC) from T1 to T2 were marked in the scaffolded condition (B = 0.53, SE = 0.17, *p* = 0.016, 95% CI [0.07, 0.99]) but were not observed in the non-scaffolded condition (B = 0.02, SE = 0.17, *p* = 1.00, 95% CI [-0.43, 0.47]), thus supporting Hypothesis 2. However, this effect did not extend from T1 to T3. Detailed regression findings are in Table 2. Additionally, cisgender women showed higher EC levels, while master’s-level clinicians exhibited lower EC than other trainees.

**Table 3:** Multilevel regressions testing the relationship between Time and Condition to the likelihood of returning

Variable	B	SE	*P*	95% CI
**Fixed Effects**				
(Intercept)	2.18	0.78	0.008	[0.57, 3.72]
**Clinician Age**	**0.04**	**0.02**	**0.011**	**[0.009, 0.07]**
Cisgender Woman	0.13	0.20	0.526	[-0.27, 0.52]
Clinician Degree: Staff MD/DO	-0.45	0.32	0.154	[-1.08, 0.17]
Clinician Degree: Staff Psychologist	-0.72	0.40	0.078	[-1.52,0.08]
Clinician Degree: MSW/MHC/MFT	-0.70	0.50	0.159	[-1.68, 0.28]
Clinician Degree: Trainee Psychiatrist	0.50	0.34	0.143	[-0.17, 1.17]
Clinician Degree: Trainee Psychologist	-0.45	0.36	0.217	[-1.17, 0.27]
**Clinician Degree: Masters Clinician**	**-1.36**	**0.59**	**0.023**	**[-2.52, -0.19]**
Clinician Degree: Other	-0.19	0.44	0.658	[-1.05, 0.67]
Study Condition - Non-Scaffolded	-0.25	0.36	0.485	[-0.96, 0.46]
Time - T2	0.09	0.31	0.767	[-0.52, 0.70]
Time - T3	-0.02	0.32	0.948	[-0.65, 0.61]
Non-Scaffolded x T2	-0.002	0.43	0.996	[-0.85, -0.85]
**Non-Scaffolded x T3**	**0.97**	**0.45**	**0.032**	**[0.08, 1.86]**
**Random Effects**				
$\tau _{00,subject}$	Rater: 0.98 CP:0.00			
$\sigma ^{2}$	1.03			
$N_{subject}$	Rater: 6 CP:49			
Observations	137			
Deviance	409.5			

Results that are statistically significant are highlighted in bold

### Raters with lived experience evaluations of clinical efficacy

Hypothesis 3 tested if both VHI training methods will effectively improve clinical efficacy and if scaffolded training would be superior to non-scaffolded training from pre- (T1) to post-training (T2 and T3). Hypothesis 3 was not supported. The result is included below:

#### Likelihood of returning for follow-up appointments

A significant interaction was observed between Time and Condition on the likelihood of patient follow-up from T1 to T3 (B = 0.97, SE = 0.45, *p* = 0.032), but not from T1 to T2 (B = -0.002, SE = 0.43, *p* = 0.996). Specifically, the likelihood of return in the non-scaffolded condition significantly increased from T1 to T3 (B = 0.95, SE = 0.33, *p* = 0.046, 95% CI [0.01, 1.89]), unlike in the scaffolded condition (B = -0.02, SE = 0.34, *p* = 1.00, 95% CI [-0.98, 0.93]), showing no significant change. No main effects were significant. Multilevel regression results are detailed in Table 3.

Additionally, clinician age positively correlated with patient return likelihood, whereas being a Master’s-level clinician was negatively associated, compared to psychologists and psychiatrist trainees.

**Table 4:** Multilevel regressions testing the relationship between Time and Study Condition in relation to Perceived Helpfulness

Variable	B	SE	*P*	95% CI
**Fixed Effects**				
(Intercept)	1.74	0.78	0.032	[0.13, 3.29]
**Clinician Age**	**0.05**	**0.02**	**0.004**	**[0.01, 0.08]**
Cisgender Woman	0.30	0.20	0.139	[-0.10, 0.70]
Clinician Degree: Staff MD/DO	-0.43	0.32	0.180	[-1.07, 0.20]
Clinician Degree: Staff Psychologist	-0.64	0.41	0.116	[-1.46, 0.17]
Clinician Degree: MSW/MHC/MFT	-0.86	0.50	0.089	[-1.85, 0.14]
Clinician Degree: Trainee Psychiatrist	0.58	0.34	0.090	[-0.10, 1.26]
Clinician Degree: Trainee Psychologist	-0.50	0.37	0.180	[-1.24, 0.24]
**Clinician Degree: Masters Clinician**	**-1.91**	**0.59**	**0.002**	**[-3.08, -0.70]**
Clinician Degree: Other	-0.02	0.44	0.963	[-0.90, 0.85]
Study Condition - Non-Scaffolded	-0.27	0.36	0.461	[-0.99, 0.45]
Time - T2	-0.02	0.31	0.948	[-0.63, 0.59]
Time - T3	-0.05	0.32	0.867	[-0.69, 0.58]
Non-Scaffolded x T2	0.04	0.43	0.919	[-0.81, 0.90]
**Non-Scaffolded x T3**	**0.92**	**0.45**	**0.044**	**[0.01, 1.82]**
**Random Effects**				
$\tau _{00,subject}$	Rater: 0.96 CP: 0.00			
$\sigma ^{2}$	1.05			
$N_{subject}$	Rater: 6 CP:49			
Observations	137			
Deviance	411.5			

Results that are statistically significant are highlighted in bold

#### Perceived helpfulness in reducing suicidal intent

A significant interaction was noted between Time and Condition on the perceived helpfulness of clinicians in reducing suicidal intent from T1 to T3 (B = 0.92, SE = 0.45, *p* = 0.044), but not from T1 to T2 (B = 0.04, SE = 0.43, *p* = 0.919). Specifically, in the non-scaffolded condition, there was a non-significant increase in perceived helpfulness from T1 to T3 (B = 0.86, SE = 0.33, *p* = 0.086, 95% CI [-0.06, 1.79]), unlike in the scaffolded condition (B = -0.05, SE = 0.33, *p* = 1.00, 95% CI [-1.00, 0.89]). No other significant effects were observed. Details are in Table 4.

Clinician age was positively related to perceived helpfulness in reducing suicidal intent. Conversely, Master’s-level clinicians were seen as less helpful compared to psychologists (staff/trainee) and psychiatrist trainees.

**Table 5:** Multilevel regressions testing the relationship between Time and Condition in relation to Overall Interview Satisfaction

Variable	B	SE	*P*	95% CI
**Fixed Effects**				
(Intercept)	73.25	14.61	<0.001	[43.46, 102.54]
**Clinician Age**	**0.86**	**0.32**	**0.011**	**[0.20, 1.50]**
Cisgender Woman	1.17	4.03	0.773	[-6.86, 9.26]
**Clinician Degree: Staff MD/DO**	**-13.60**	**6.46**	**0.041**	**[-26.65, -0.75]**
Clinician Degree: Staff Psychologist	-12.91	8.18	0.121	[-29.36, 3.43]
Clinician Degree: MSW/MHC/MFT	-15.88	10.16	0.126	[-36.23, 4.55]
Clinician Degree: Trainee Psychiatrist	8.01	6.93	0.253	[-6.01, 21.76]
Clinician Degree: Trainee Psychologist	-12.63	7.46	0.097	[-27.80, 2.25]
**Clinician Degree: Masters Clinician**	**-35.96**	**12.00**	**0.004**	**[-59.65, -11.33]**
Clinician Degree: Other	-5.83	8.94	0.517	[-23.90, 11.93]
Study Condition - Non-Scaffolded	-9.08	7.10	0.203	[-23.28, 5.12]
Time - T2	4.79	5.93	0.422	[-6.99, 16.54]
Time - T3	-0.51	6.24	0.935	[-12.86, 11.90]
Non-Scaffolded x T2	3.62	8.33	0.665	[-12.86, 20.18]
**Non-Scaffolded x T3**	**19.39**	**8.76**	**0.029**	**[1.80, 36.82]**
**Random Effects**				
$\tau _{00,subject}$	Rater: 217.79 CP:14.14			
$\sigma ^{2}$	394.14			
$N_{subject}$	Rater: 6 CP: 49			
Observations	138			
Deviance	1234.2			

Results that are statistically significant are highlighted in bold

#### Overall interview satisfaction

A significant interaction was noted between Time and Condition on the overall interview satisfaction from T1 to T3 (B = 19.39, SE = 8.76, *p* = 0.029), not seen from T1 to T2 (B = 3.62, SE = 8.33, *p* = 0.665). Specifically, satisfaction increased significantly in the non-scaffolded condition from T1 to T3 (B = 18.88, SE = 6.32, *p* = 0.032, 95% CI [0.95, 36.80]), unlike in the scaffolded condition (B = -0.51, SE = 6.45, *p* = 1.00, 95% CI [-18.79, 17.77]). No other significant effects were noted. Detailed regression results are in Table 5.

Clinician age positively impacted overall satisfaction, while MD/DO or Master’s-level credentials were linked to lower satisfaction.

## Discussion

This work explores the impact of scaffolded instructions and reminders on VHI training designed to help clinicians manage their negative emotional responses and engage in verbal empathic communication with virtual patients representing people at risk for suicide.

The findings identified VHI training with post-interview feedback, both with and without scaffolding, significantly decreased clinician participants’ NER, as self-quantified by the TRQ-SF survey, after two training sessions but not immediately after the first training session. This finding aligns with previous literature indicating that multiple training sessions are needed to decrease NERs in healthcare professionals. Previous research has provided evidence for the impact of using the mindfulness-based stress reduction program, which is delivered in multiple sessions, to help healthcare professionals manage their emotions [51]. The mindfulness-based stress reduction program is a structured group program that needs to be delivered in eight sessions, one session per week [52]. The reason to have multiple sessions is that the development of the ability to sustain attention to negative emotions is gradual and progressive and requires regular practice [52, 53]. Therefore, the finding implies that multiple VHI training sessions are needed to train clinicians’ skills in managing NERs.

Our findings show that scaffolded instructions were more helpful in improving clinician participants’ verbal empathic communication in one training session than post-interview feedback alone. The result is aligned with the previous research that integrating scaffolded instructions into virtual human interactions helped clinicians provide less low-empathy level and more medium- and high-empathy level responses in the interaction with the virtual patient [32]. Furthermore, this study also shows that the benefits of using scaffolded instructions in VHI to improve verbal empathic communication are enduring. Participants’ verbal empathic communication did not decrease in T3 in the scaffolding condition, although the scaffolded instructions were only provided in T1 and T2. This finding aligns with previous research by Finn et al., indicating that scaffolded feedback led to more resilient corrections over a delay interval than standard feedback [31]. This finding could be generalized to other contexts where VHIs are used in behavioral training, integrating scaffoldings into the VHIs to enhance the training impact.

Given that the findings for Hypothesis 2 regarding verbal EC indicated that scaffolded training was superior to the one without scaffolding, the findings regarding clinical efficacy, as rated by raters, were unexpected. In this study, raters with lived experience of suicide attempts watched the recorded interaction videos showing clinician participants’ faces, read the interaction transcripts, and rated the likelihood of returning for follow-up appointments and overall interview satisfaction. Therefore, raters could evaluate both verbal and nonverbal channels. Some comments we received from raters also proved that they evaluated both verbal and nonverbal channels.

*“I liked this clinician’s facial expressions when they asked questions. They were very emotionally expressive, so I felt the clinician came across as caring”.*

*“I had mixed feelings about this clinician. With their facial expressions and attitude, they came across as empathic, and they did ask a number of open-ended questions that showed interest in me, but at the same time, they kept harping on asking me if I wanted to hurt myself, which was not helpful”.*

*“This clinician came across as somewhat friendly as well as inquisitive in tone but did not smile during the session, which would have been helpful. Their voices also felt a bit monotone, and their facial expressions were not too expressive, so this made them seem kind of indifferent and less caring. I truly liked these questions that were asked: “Is there anything else on your mind?” “What is it that you’re hoping for today?” “What would you like to look different in your life?” and “What helps you feel better?” These are all very open-ended questions that require more than a short or yes/no response”.*

In this study, we provided scaffolded instructive descriptions and reminders to reflect NERs using popup-style dialog boxes. The use of dialogs may hinder users’ experience and thus induce some verbal or nonverbal behaviors that make raters not score clinicians’ interactions with virtual patients significantly higher in T3 than in T1. Our preliminary results show that trainees’ dissatisfaction with the VHI technological limitations results in negative facial affective behaviors (such as anger) detected by the NOLDUS face reader, which may be noticeable to the raters and may lower the clinical efficacy of scaffolding which is perceived as more difficult technologically [32, 54]. This may explain why scaffolded training was superior in improving clinicians’ verbal empathic communication skills but did not help in improving clinician clinical efficacy rated by raters. Clinicians in the scaffolded training condition might be able to be verbally empathic but show negative facial expressions or tone of voice resulting from dissatisfaction with the interaction experience. Clinicians’ negative facial expressions or tone of voice might affect raters’ ratings, although the clinician tried to express empathy verbally. Previous literature shows that people rely more heavily on nonverbal cues to decode messages when verbal and nonverbal cues are incongruent [55–57]. Future work could explore alternative methods for delivering real-time scaffolded instructions during interactions, such as through interaction metaphors like the virtual human’s facial expressions or body gestures, rather than relying solely on popup-style dialog boxes. This approach aims to minimize the negative impact on the interaction experience. Moreover, in this study, scaffolded instructions were integrated into the interaction to enhance verbal empathic communication. Future research should investigate how to effectively provide real-time scaffolded instructions for nonverbal cues to express empathy, including facial expressions, gestures, and vocal tones.

Another reason scaffolded training improved clinicians’ empathic communication, measured by the ECCS, but not their clinical efficacy (as rated by raters), was possibly due to the inherent limitations of structured communication trainings [58]. The traditional focus on specific behavioral skills has been criticized, with a shift towards valuing the essence of communication tasks like forming connections and expressing that one cares [59–61]. Communication complexity, especially in contexts like consultations and relationship building, challenges the efficacy of behavioral coding schemes for full evaluation [58]. Expert-designed schemes might not align with patient values, suggesting that expert-approved communication improvements don’t always meet patient needs [62–65]. However, ignoring patient cues during empathic opportunities will be consistently damaging across all consultations [16, 17, 58]. The APC (Acknowledge, Pursue, Confirm) scaffolding, based on the ECCS, can remind clinicians to acknowledge and confirm patients’ feelings [32]. Therefore, integrating VHIs with scaffolded instructions offers a valuable approach to enhancing clinicians’ empathic communication skills.

A limitation of this study is that the interactions are not counterbalanced. Participants in the non-scaffolded condition interacted with Cynthia at T1, Bernie at T2, and Denise at T3. Participants in the scaffolded condition interacted with Denise at T1 and were randomly assigned to interact with Cynthia at T2 and Bernie at T3 or with Bernie at T2 and Cynthia at T3. We analyzed average ECCS scores across the three virtual humans (collapsing across T1, T2, and T3). A repeated-measures ANOVA indicated that there were differences in ECCS average scores across the three virtual humans (F[2, 82] = 4.98, *p* = 0.009). Specifically, with a Bonferroni correction, clinicians had higher empathic communication scores with Bernie (M = 2.73, SD = 0.70) than they did with Cynthia (M = 2.26, SD = 0.67, *p* = 0.001); there were no differences between Denise (M = 2.44, SD = 0.79) and either Cynthia (*p* = 0.296) or Bernie (*p* = 0.366).

Bernie was always either second or third in order, so this may reflect that clinicians had received practice and feedback with prior virtual humans. In contrast, Cynthia was always first in the scaffolded condition. Future work needs to investigate whether this is an order effect or differences between virtual humans.

## Conclusion

In this study, we aimed to integrate scaffolded instructions and reminders into virtual human interactions to improve clinician participants’ verbal empathic communication, remind them to reflect on their negative emotions and improve their clinical efficacy. Virtual human interaction training sessions, both with and without integrated scaffolded instructions and reminders, are helpful for clinicians in managing their negative emotional responses and increasing verbal empathic communication. However, incorporating scaffolded instructions and reminders is more helpful for participants to engage in verbal empathic communication but not to increase perceived patient satisfaction and the likelihood of returning for follow-up appointments. The pop-up dialogs might hinder participants’ interaction with the virtual patient and induce negative emotional responses in the interaction.

## Data Availability

The data that support the findings of this study are available from the corresponding author upon reasonable request.

## Abbreviations

VHI: Virtual Human Interaction
EC: Empathic Communication
EO: Empathic Opportunity
ZPD: Zone of Proximal Development
NER: Negative Emotional Responses
CE: Clinical Efficacy
VPF: Virtual People Factory
TRQ-SF: The Therapist Response Questionnaire-Suicide Form
CP: Clinician Participant
VP: Virtual Patient
ECCS: Empathic Communication and Coding System
